# The resurgence of syphilis in high-income countries in the 2000s: a focus on Europe

**DOI:** 10.1017/S0950268819000281

**Published:** 2019-03-13

**Authors:** G. Spiteri, M. Unemo, O. Mårdh, A. J. Amato-Gauci

**Affiliations:** 1 European Centre for Disease Prevention and Control, Stockholm, Sweden; 2 Department of Laboratory Medicine, Microbiology, Faculty of Medicine and Health, WHO Collaborating Centre for Gonorrhoea and other STIs, Örebro University, Örebro, Sweden

**Keywords:** Epidemiology, surveillance, syphilis (*T. pallidum* infection)

## Abstract

Syphilis can cause severe complications and sequelae. Following a decrease in reported cases in European Union/European Economic Area (EU/EEA) and other high-income countries in the 1980s and 1990s as a result of the HIV epidemic and ensuing changes in sexual behaviour, trends started to increase in the 2000s in a number of EU/EEA Member States with higher rates among men and a large proportion of cases reported among men who have sex with men (MSM), particularly HIV-positive MSM. Trends in EU/EEA Member States vary however with some countries continuing to report decreases in the number of reported cases (mostly in the Eastern part of EU/EEA) whereas many Western European countries report increasing numbers of cases. Increasing rates among women, although still relatively low, have been observed in a number of countries leading to concerns around mother-to-child transmission of syphilis and congenital syphilis. Similar overall trends are observed in other high-income countries with the exception of Japan where rates among heterosexual men and women have been rising at alarming levels. Control of syphilis requires use of comprehensive, evidence-based strategies which take into account lessons learned from previous control efforts as well as consideration of biomedical interventions.

## Introduction

Syphilis is caused by the spirochaete *Treponema pallidum* subspecies pallidum. The infection is mainly sexually transmitted, except for transfusion of blood or blood products and congenital syphilis (mother-to-child transmission (MTCT) of syphilis), which is transmitted vertically during pregnancy. Following infection, syphilis may present as a chancre (primary syphilis) 10–90 days (average 3 weeks) after exposure [1]. If untreated, the chancre will heal, but within a few weeks or months symptoms of secondary syphilis may appear. Symptoms may vary and include rashes, alopecia, condylomata lata as well as unspecific symptoms including malaise, sore throat, weight loss and low-grade fever. Symptoms of secondary syphilis disappear within some weeks, even without treatment. Latent syphilis then follows where patients do not show any symptoms but may remain infectious for up to a year following infection [2]. Latent infection may last for decades; if untreated, tertiary syphilis may develop with symptoms depending on the organ involved. Tertiary complications are rare at the present time, likely due to the impact of intermittent antibiotic use. Neurosyphilis, however, can still be observed and may present at any stage of syphilis. There are many diverse clinical manifestations of neurosyphilis [1]. Benzathine penicillin G is the recommended treatment for syphilis and no bacterial resistance has been confirmed yet [3–6].

Historically, prior to the advent of penicillin, syphilis caused a significant public health burden with rates of infection in the general population of some countries of 5–10% [7]. Syphilis was an important cause of neurological and cardiovascular complications and congenital syphilis was a significant cause of perinatal mortality. The rates of syphilis rapidly decreased following the widespread use of penicillin in the 1940s [8, 9]. Since then, syphilis has followed social trends with, for example, increases related to the sexual liberation of the 1960s, the crack-cocaine epidemics in the USA in the late 1980s and 1990s as well as decreases following the HIV epidemic in the 1980s and the subsequent changes in sexual behaviour [10, 11]. However, since the 2000s, syphilis rates have again been increasing in developed countries, with rates rising most rapidly among men who have sex with men (MSM), but also increasing in other population groups [12]. The interaction with HIV co-infection, changes in sexual behaviour following wider availability of effective antiretroviral HIV treatment, the changing means and ease of finding sexual partners as a consequence of Internet and mobile dating applications have increased the complexity of the syphilis epidemiology, and particularly its control.

The aim of this review was to describe the recent epidemiology of syphilis in high-income countries, focusing on the European Union and the European Economic Area (EU/EEA) and to highlight emerging issues in syphilis epidemiology and control. This was done by reviewing syphilis surveillance data published by the European Centre for Disease Prevention and Control (ECDC), including surveillance reports and online databases. Additional key publications supporting the surveillance results were identified by reviewing the peer-reviewed published literature. A similar approach was used for other high-income countries and for MTCT of syphilis. Emerging issues were identified following discussion between coauthors and through reviewing peer-reviewed publications, where available from countries in the EU/EEA, but otherwise from other high-income countries.

## Present situation: EU/EEA

**Fig. 1.**
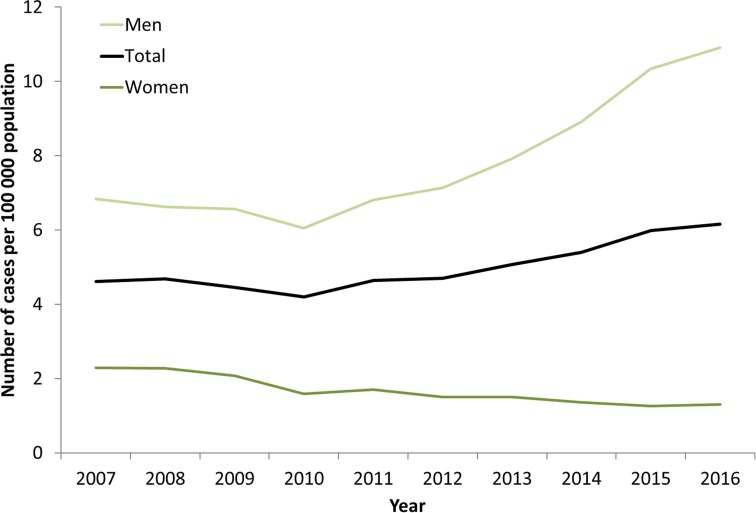
Rate of confirmed syphilis cases per 1 00 000 population by gender and year, EU/EEA countries reporting consistently, 2007–2016 (from [13]).

*Source*: Country reports from Bulgaria, Cyprus, the Czech Republic, Denmark, Estonia, Finland, Germany, Ireland, Italy, Latvia, Luxembourg, Malta, Norway, Poland, Portugal, Romania, Slovakia, Slovenia, Sweden and the UK.

**Fig. 2.**
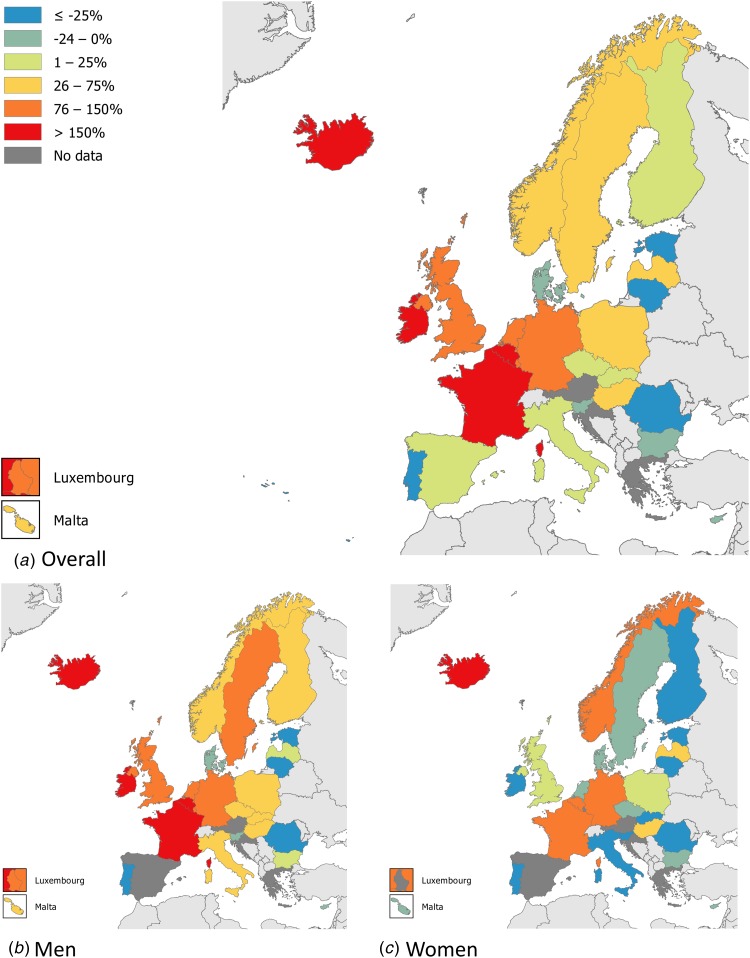
Changes in number of reported syphilis cases overall and among men and women, between 2010 and 2016 (adapted from [14]).

Syphilis surveillance data reported at the EU/EEA level showed an overall decreasing trend from 8.4 cases per 1 00 000 population in 2000 to 4.4 in 2010, followed by an increase up to 6.1 cases per 1 00 000 in 2016 when 29 365 cases of syphilis were reported in 28 EU/EEA countries (Fig. 1) [12, 13]. This overall trend, however, masks the heterogeneity in trends observed across different countries. Syphilis rates in the Eastern part of the EU/EEA peaked around the late 1990s and early 2000s at very high rates (e.g. Latvia (in 1996): 126 per 1 00 000 and Romania (in 2002): 55 per 1 00 000 persons) and decreased thereafter. Rates in most Western and Central EU/EEA countries started from very low levels in the early 2000s and have been increasing steadily since then at different rates, although they still have not reached levels seen in Eastern Europe in the early 2000s (Fig. 2). At the EU/EEA level, there are gender differences, with rates among women decreasing overall since the peak in 1997. In contrast, rates among men decreased from 2005 to 2010 but have since increased to the highest rates reported since surveillance started at EU/EEA level (1990) (Figs 1 and 2 and [14]). These gender-specific patterns also vary by region within the EU/EEA, with large increases in rates reported among women in the Eastern part in the early 2000s, whereas Western and Central EU/EEA countries reported lowest rates among women in the early 2000s. Since then, cases among women in Western and Central EU/EEA countries such as the Czech Republic, Denmark, France, Germany and the UK have increased although rates are still lower than in Eastern EU/EEA countries (Fig. 2). Trends among men in Eastern EU/EEA countries followed similar patterns as for women, peaking in the mid-1990s and early 2000s, then decreasing and stabilising since 2010 [12–14].

**Fig. 3.**
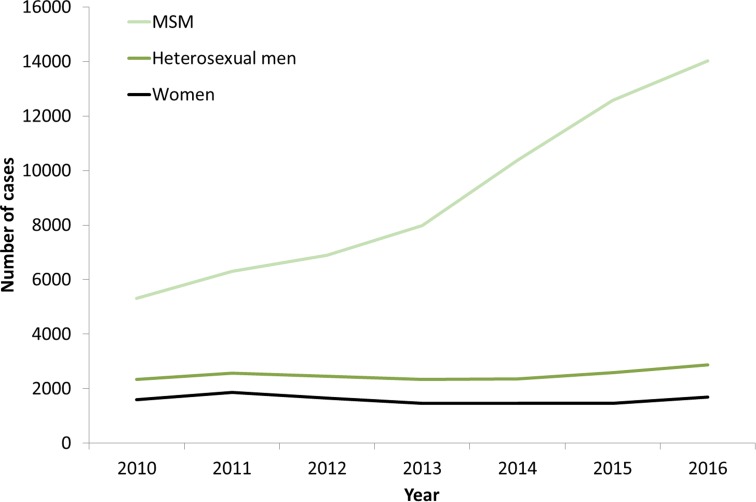
Number of confirmed syphilis cases by gender, transmission mode (sexual orientation) and year, EU/EEA countries reporting consistently, 2010–2016 (from [13]).

*Source*: Country reports from the Czech Republic, Denmark, Finland, France, Germany, Hungary, Iceland, Ireland, Latvia, Lithuania, Malta, the Netherlands, Norway, Portugal, Romania, Slovakia, Slovenia, Sweden and the UK.

The increases in syphilis cases among men in the EU/EEA have been driven by transmission among MSM (Fig. 3) [12–17]. This is clearly the case for Western EU/EEA countries; however, although data are limited, similar observations have been reported from certain cities in Eastern and Central EU/EEA, for example, Gdansk and Warsaw in Poland [18]. In addition, syphilis seropositivity among MSM in Bucharest was over 16% in a respondent-driven sampling study in 2012–2013, indicating a significant burden of syphilis among among MSM there [19]. The number of cases reported among MSM in the EU/EEA has more than doubled (164% increase) from 2010 to 2016 (Fig. 3). Increases in number of infections among MSM have been reported among HIV-negative and HIV-positive MSM, with some studies reporting greater increases among HIV-positive MSM [17]. Trends among heterosexual men at the EU/EEA level have, on the other hand, remained stable overall, although slight increases have been reported in 2015 and 2016 [13].

## Other high-income countries

Similar trends as in Western EU/EEA countries have been described in high-income countries outside of Europe. In the USA, primary and secondary syphilis cases increased by 74% from 2003 to 2017, from 5.5 cases per 1 00 000 persons to 9.5, continuing an increasing trend since a nadir in 2001. Men accounted for 88% of syphilis cases in the USA in 2017 and 58% of all cases were among MSM [20]. In Canada, infectious syphilis rates increased from 5.0 to 9.3 per 1 00 000 persons from 2010 to 2015, an increase of 86%. The increase in Canada was more pronounced among males (90% compared with 28% among females) and males accounted for 94% of cases of infectious syphilis in 2015 [21]. In Australia, the overall syphilis notification rate increased by 107% from 6.9 cases per 1 00 000 persons in 2012 to 14.3 in 2016. Notification rates in Australia have increased among both males and females during this time with increases being proportionately more marked among females. However, males accounted for 87% of infectious syphilis cases in 2016. Notification rates were 5.4 times higher among the Aboriginal and Torres Strait Islander population than in the non-indigenous population. The increase from 2012 to 2016 was also higher among the Aboriginal and Torres Strait Islander population compared with the non-indigenous population (193% *vs.* 100% increase, respectively) [22]. In Japan, syphilis cases had increased in 2016 to levels not seen since the 1970s [23]. Although initially the increase was seen mostly among MSM [24], since 2013 the number of reported syphilis cases in Japan among heterosexual men and women have increased dramatically and each group accounted for more diagnoses in 2016 than MSM [23] leading to concerns about the re-emergence of congenital syphilis.

## MTCT of syphilis

Apart from the increases in MSM, the USA, Canada, Australia and some Western EU/EEA countries have observed an increase in infections among heterosexual women. Although absolute rates among women continue to be low, these increases are worrying due to the risk of MTCT of syphilis. Indeed, there have been recent increases in cases of congenital syphilis in the USA and Japan [23, 25]. In Europe, congenital syphilis rates have decreased in recent years although cases are likely under-reported [26]. The countries reporting the largest numbers of cases have seen the largest decreases, for example, Bulgaria and Romania [14]. Despite this, cases are still reported in many EU/EEA countries (10 of the 23 reporting countries in 2016) and there have been recent reports of cases from the UK among mothers who screened negative in the first trimester of pregnancy [27]. An ecological analysis found that these cases occurred in areas with increases in syphilis among women and MSM and relatively high proportion of behaviourally bisexual MSM which might have led to bridging of the syphilis epidemic from MSM to heterosexuals [28]. These cases emphasise the need for strengthened antenatal syphilis screening, subsequent appropriate treatment and follow-up of screening-positive cases during the phase of elimination of MTCT of syphilis and HIV infections as declared by the World Health Organization [29].

## Ocular syphilis

Recent reports of clusters of ocular syphilis in the USA [30] created concerns of possible oculotropic strains. However, preliminary molecular epidemiological typing investigations have so far not confirmed the presence of a single oculotropic strain [31]. It is still unclear whether the increase in ocular syphilis is a result of increased recognition and subsequent testing of ocular manifestations, a real increase in the proportion of cases with ocular manifestations, and/or a consequence of the increase in syphilis cases being observed among MSM. A report describing observations from eight jurisdictions in the USA showed that reported ocular syphilis cases from 2014 to 2015 had an epidemiology that appeared to reflect that of syphilis in the USA in terms of predominance among men, proportion among MSM and percentage HIV-positive [32]. Authors from UK also report that trends of ocular syphilis in Manchester have increased in line with the epidemic of early syphilis. Of the 34 cases in their 2002–2016 case series, almost all were male (94%), 75% were MSM and 29% (all men) were HIV-positive. They estimate a risk of 1% for ocular involvement among those with early syphilis infection [33].

## Reinfection

One aspect of the recent increases in syphilis among MSM observed in some countries has been the large proportion of repeat infections. In Antwerp, Belgium, for example, the majority of syphilis episodes from 1992 to 2012 were reported among MSM reporting more than one syphilis episode [34]. Observations from San Diego county in the USA also showed a substantial proportion of repeat syphilis [35]. HIV-positive MSM, those with 10 or more partners in the interval when syphilis was most likely acquired, as well as MSM who fail to attend for follow-up of the initial episode have been associated with a higher risk of repeat infection [34, 36].

## Outbreaks

Despite the increase in reported syphilis across Europe, defined syphilis outbreaks are not often described in the EU/EEA. Key features associated with reported outbreaks include transmission among MSM, involvement of MSM often showing high prevalence of co-infections including HIV and hepatitis C, large number of recent sexual partners, including new and unknown partners, per time unit, as well as the use of geospatial networking apps to source partners [37–40]. The increasing use of social media applications for finding sexual partners, particularly among MSM, has been frequently associated with increases in sexually transmitted infection (STI) despite limited evidence. Geospatial apps and websites, however, also provide an opportunity to reach key groups with public health messaging, although such campaigns need to be effectively designed to allow for an impact on outbreak control [39, 41, 42].

Newer epidemiological methods such as space-time clustering have been used to investigate syphilis epidemiology in the UK and the Netherlands. These identified previously undetected outbreaks among HIV-positive and HIV-negative MSM [15, 43]. Such analyses could support syphilis control, management and guide public health interventions.

## Syphilis treatment

Syphilis treatment continues to be based on benzathine penicillin G, and decades of clinical experience, clinical case studies, laboratory considerations and biological plausibility support its effectiveness [4, 6, 44]. Use of the appropriate preparation is essential as some forms of penicillin do not reach sequestered sites in the body and there have been reports of inadvertent use of inappropriate penicillin formulations [45]. Data on the effectiveness of alternative regimens are limited. The European syphilis management guideline recommends intramuscular procaine penicillin for 10–14 days as second-line therapy option if benzathine penicillin G is not available with additional options, such as doxycycline, ceftriaxone or azithromycin, in case of bleeding disorders, penicillin allergy or refusal of parenteral treatment [44]. All three alternatives are effective; however, a recent meta-analysis found that doxycycline/tetracycline recipients had a higher treatment failure rate than those treated with benzathine penicillin G [46–50]. In addition, azithromycin resistance and treatment failures have been described in the USA, Europe, Australia, China and elsewhere and azithromycin should therefore only be used as a last resort and when treatment can be followed up closely [6, 44, 51].

## Discussion

The main epidemiological trends of syphilis in the EU/EEA in recent years are driven by increased cases among MSM. This observed increase among MSM is likely multifactorial and linked to increased numbers of partners, particularly casual partners, sero-adaptive behaviours and reduced condom use particularly among HIV-positive MSM [16, 52–54]. In more recent years, it is likely that the impact of effective HIV treatment as prevention and, even more recently, the introduction of HIV pre-exposure prophylaxis (PrEP) have led to increased risk behaviours and consequently syphilis transmission [55–57]. Control of syphilis, as well as other STIs, among MSM is challenging in the era of PrEP and highly active antiretroviral treatment [58].

Apart from the challenges in controlling syphilis among MSM, the rapid change in the epidemiology of syphilis in Japan [23], with increased infections among heterosexual men and women together with the increase in MTCT of syphilis, highlights another significant public health issue that might also affect many additional high income countries. Indeed, the USA and a number of EU/EEA countries have already started observing increases in syphilis rates among women and in certain cases increases in MTCT of syphilis. Effective antenatal screening programmes, including appropriate screening and subsequent treatment and follow-up of screening-positive cases, are implemented in most parts of the EU/EEA and some countries already implement repeat screening in the third trimester of pregnancy [59]. In areas with ongoing outbreaks where repeat screening is not implemented, women at risk should be considered for such a second screening opportunity during the third trimester or even at delivery. In such situations, rapid serological point-of-care tests could also be considered for use when a pregnant woman who has not been screened earlier, with unknown screening results or who has been exposed since her screening test attends for delivery [60]. Unfortunately, women at risk can be difficult to identify [28]. The EU/EEA rate of MTCT of syphilis is already below the World Health Organization elimination target (50 per 1 00 000 live births). With the re-emergence of syphilis, however, attention should be given to monitoring the performance of antenatal screening programmes and to increasing awareness among healthcare professionals. In addition, public health agencies should consider evaluating confirmed MTCT of syphilis in detail to identify ways of optimising antenatal programmes to prevent future infections. Standardised case definitions need to be used to ensure that all episodes of MTCT of syphilis are captured.

Syphilis control requires a multi-targeted approach using all available tools, and also new opportunities. Existing tools, such as primary and secondary prevention interventions, including enhanced awareness in the general population, persons at risk and among health care professionals, facilitation and expansion of testing and subsequent treatment and follow-up of patients, and partner notification need to be implemented as widely as possible [61]. Many of these interventions could be further enhanced through the use of geospatial apps and websites as mentioned earlier. Focusing particularly on regular STI testing of MSM, particularly when on PrEP, as recommended in guidelines, is essential [4, 6, 61, 62]. Expansion of syphilis testing can be supplemented through community-based initiatives, successful examples of which have been implemented in the EU/EEA [63, 64]. Although many community-based settings focus mostly on HIV testing, syphilis testing should be routinely included in the context of high prevalence rates. Such expansion of testing could be established through rapid point-of-care testing technologies including combined HIV and syphilis testing methodologies once these have been thoroughly validated [65–67].

Recently, post-exposure prophylaxis with doxycycline for syphilis has been investigated and found to be effective in reducing syphilis [68, 69]. Further research is needed to investigate the use of such an intervention in the context of core transmitter groups with high incidence of infection and re-infection. Although there are concerns around the development of antimicrobial resistance in *T. pallidum* and particularly in other bacterial species, the use of post-exposure prophylaxis for a limited period of time in groups with a high intensity of syphilis transmission as part of a comprehensive prevention intervention as suggested by Molina *et al*. warrants further investigation [68]. In addition, an effective oral therapy for syphilis would be valuable and a randomised controlled clinical trial evaluating cefixime for treatment is currently being planned [70]. Availability of alternative treatment options are particularly important due to shortages of benzathine penicillin G reported in many countries in recent years. These shortages have been in part linked to producers of the active product ingredient exiting the market as a consequence of the low market price and high production costs [71]. Despite the potential of alternative treatments, securing the availability of benzathine penicillin G is essential for global syphilis control.

The limited data and lack of reported syphilis outbreaks in many parts of Europe in a context of increasing trends highlight the challenges in prioritising STI surveillance and public health response in many countries. There is scope for a wider response to the resurgence of syphilis in Europe. In the USA, plans for syphilis control were launched in 1999 and 2006 with the 2006 plan proposing a reduction in primary and secondary syphilis, a reduction in congenital syphilis and reduced racial disparities by 2010. Three goals were proposed: investment in and enhancement of public health services; prioritisation of evidence-based, culturally competent interventions; and creating accountable services and interventions [72]. The targets were not reached and further increases in syphilis and MTCT of syphilis have since been reported. The experience from the USA provides a number of lessons which are useful for most countries trying to deal with this epidemic, including improving access to care, expanding partnerships (e.g. with other disease programmes), tailoring control activities to the epidemic and ensuring programme evaluation [73].

The syphilis epidemic should however not be taken as an isolated issue. Other STIs are on the increase both among MSM and heterosexuals [58]. Increasing trends and/or outbreaks of gonorrhoea, lymphogranuloma venereum, and sexually transmitted shigellosis and hepatitis A have been reported in high-income countries around the globe over the last few years [74–77], indicating a need to focus more widely on improving sexual health rather than targeting specific infections. An integrated cross-disease approach would also result in synergies with other public health programmes which are often better resourced, such as HIV control.

One of the key challenges in syphilis control in EU/EA is the diversity in populations, resources, healthcare systems and public health services including surveillance. A one-size-fits-all approach is therefore unlikely to be effective. The lessons from the experience of the syphilis control plans in the USA certainly need to be borne in mind. The paucity of data on the syphilis epidemic from some parts of Europe is an additional point that needs to be kept in mind for an effective syphilis response in Europe. Ensuring that surveillance and other data are available at sufficient quality across EU/EEA is essential in order to guide an effective response. A better understanding of the key groups affected by syphilis across Europe, including (but not only) through better collection of data on sexual orientation, would allow for better understanding of the drivers of the epidemic, particularly in terms of socio-economic factors which clearly have an impact on syphilis epidemiology.

## Conclusion

The resurgence of syphilis over the last decade in high-income countries of EU/EEA has clearly been driven by epidemics among MSM. Although this is a burden which needs to be addressed through specific interventions, the increase in cases among women, combined with increases in some settings of MTCT of syphilis, are worrying due to high risk of still birth and many additional severe complications and sequelae in affected newborn infants. Control of the current syphilis epidemic will not be achieved through any single intervention. Approaching syphilis control through a comprehensive strategy which applies all available evidence-based prevention interventions, including increased condom use, regular testing, behavioural interventions, together with effective treatment as well as further investigation of emerging biomedical tools and development of novel prevention approaches adapted to current social, behavioural and epidemiological contexts provides the best chance for success.
